# A Biomechanical Analysis of Lower Limb Movement on the Backcourt Forehand Clear Stroke among Badminton Players of Different Levels

**DOI:** 10.1155/2019/7048345

**Published:** 2019-01-14

**Authors:** Xiaoxue Zhao, Shudong Li

**Affiliations:** Faculty of Sports Science, Ningbo University, Ningbo 315211, China

## Abstract

Most of the previous studies have been focused on the upper limb biomechanical characteristic in the clear stroke among different level badminton players, but research on the lower limb is limited. The aim of this study is to explore the lower limb kinematics and foot pressure in the backcourt forehand clear stroke among badminton players to give theoretical reference in teaching and answer the questions occurring in the process of learning the actions. Ten professional badminton players (PP) and ten amateur players (AP) were recruited in this study. Plantar pressure analyses indicated that both the PP and the AP were in contact with the ground over the forefoot without the midfoot and heel. The work suggests that when designing professional badminton sports shoes, the designers should focus on strengthening footwear resistance in the metatarsal and forefoot area, especially the first metatarsal area, to meet the requirement of the movement demand and take the badminton movement characteristics in different regions of the design. The peak ankle dorsiflexion, eversion, and internal rotation angle levels of the AP are lower than those of the PP. It is important for the AP group to enhance their ankle strength to prevent injury and improve performance.

## 1. Introduction

Badminton is one of the most popular sports in the world [1] and is a fast racket game characterized by high-intensity, intermittent actions [2]. The badminton players' performance is determined by the relationship of speed, agility, flexibility, shoulder strength, explosive strength, and muscular endurance [1]. The badminton forehand overhead techniques could be divided into three strokes: drop, clear, and smash [3]; the stroke can be divided into clear, drop, smash, block, lift, push, net, and dab [4]. The forehand overhead stroke is regarded as the fundamental to play badminton [5]. The clear strokes are among the most common strokes in badminton which account for 14~16% of the total stroke of the male players [6]. Clear strokes provide the basis of playing the shuttle from the players' own backline to the opponent's backline and are an overhead shot with a flat (offensive clear) or rising trajectory (defensive clear) towards the back of the opponent's court [1]. A good lift shot, like the type of clear stroke, which reaches the baseline of the rear court, sometimes helps the player to win the rally by causing misjudgement by the opposition player and allows the player to get more time to prepare for the next shot [4]. There is a lack of scientific research in badminton, including biomechanical investigations of stroke techniques [7].

Stroke preparation requires considerable skill for playing the shuttlecock around the full court [1, 8]. Players are required to take quick changes of direction, jumps, lunges at the net, and rapid arm movements from a variety of postural positions [9]. The footwork is the most fundamental skill in daily training and race for badminton players [10] and plays a crucial role in skill proficiency [11–13]. Footwork enables players to move into the best position by supporting a good balance and body control [14]. The hitting actions used in badminton are governed by reasonably simple biomechanical principles [6]. The overarm throwing and hitting actions used in badminton are characterized by a proximal-to-distal evolution of the movement kinematics [3]. The most frequently used footstep in the backcourt was the driving-turning (one step) for both male and female players [15]. Highly ranked youth badminton players seem to have a different stroke technique compared to lower-ranked players [7]. Different level badminton players could have significant difference on the plantar pressure characteristics when accomplishing this footstep [16]. Although there are many research articles about badminton especially on the smash and lunge step, the footwork characteristics about the backcourt forehand clear stroke technique among the badminton players are not comprehensively understood as yet. So in this study, we desire to further analyze this footstep at the special stroke technique, the backcourt forehand clear stroke. It was hypothesized that significant difference of the lower limb kinematics and foot pressure existed among different level badminton players.

## 2. Methods

### 2.1. Subjects

A total of 20 right-handed male college students participated in this study. Ten professional badminton players (ages: 23.69 ± 2.4 years; height: 182.54 ± 5.2 cm; mass: 72.53 ± 2.4 kg; badminton-playing years: 11.3 ± 1.6 years) and ten amateur players (ages: 22.48 ± 1.9 years; height: 181.74 ± 5.6 cm; mass: 71.39 ± 3.15 kg; badminton-playing years: 5.3 ± 1.4 years) were recruited in our experiments. There were no significant differences in demographic data between these two groups. For the professional badminton players (PP), they all have national badminton athlete certificates of the second level. For the amateur players, they play badminton at least twice a week. Both were right-handed and with right dominant leg. All subjects were free from lower limb injuries and surgery treatment for at least six months before the experiment and were in good health. Informed consents were obtained from all participants with their signature before participating in this test. This research was also approved by the Ethics Committee of Ningbo University.

### 2.2. Protocol

**Figure 1:**
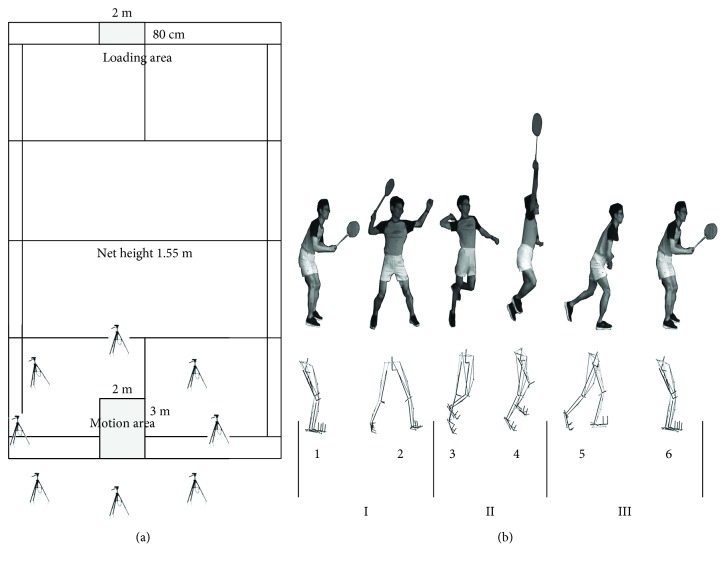
(a) A simulated badminton court in our laboratory including the eight camera positions, motion area, and shuttle loading area. (b) The detailed action diagram of the backcourt forehand clear stroke; the upper image shows one elite participant's whole body and racket movement; the lower image shows the simulation of his lower limb movement in the Vicon system.

This study was conducted in the laboratory of the Research Academy of Grand Health at Ningbo University from November of 2017 to June of 2018. They were filmed using rackets and shoes provided by our laboratory to reduce the error in the experiment. Participants were instructed to perform each test at a predefined starting point then back to the same point after contact with the shuttlecock from a standardized initial position [17]. After contact with the shuttlecock, the participants were asked to return to their starting positions at their quickest controllable speed as the racket sports require athletes to process information in a time-constrained environment [18]. Before the test, the participants were asked to perform fifteen-minute warming up and familiarization of the experiment environment. At the actual test trial, each participant was asked to perform the backcourt forehand clear stroke for six successful actions collected, as deemed by the team coach.  Figure 1 showed the footstep of the backcourt forehand clear stroke, the camera positions, and playing area according to the previous studies and the coaches' guidance [19, 20]. Participants were provided with 30-second rests between trials to minimize fatigue. As we mainly aimed to analyze the action of the lower limb, we could divide the whole backcourt forehand clear stroke into 3 phases: phase I indicating the starting step of the right leg, phase II representing the stroke motion, and phase III illustrating the backing step into the starting position. In detail, this action was characterized in 6 stages including the starting position (stage 1), the landing step of the right leg (stage 2), the right foot leaving the ground (stage 3), the stroke moment (stage 4), the right leg coming back to the starting position (stage 5), and the left leg returning to the starting position (stage 6). In this study, we mainly explore the first right leg stance phases (stage 2 and stage 3).

### 2.3. Data Collection

The Novel Pedar system (Germany) frequency at 100 Hz was used to record the plantar pressure data of subjects when they were performing the backcourt forehand clear stroke actions. Meantime, an 8-camera Vicon three-dimensional infrared motion capture system (Oxford Metrics Ltd., Oxford, UK) at 200 Hz was set up to collect the kinematics data of the lower limb. Inverse dynamics analyses were used to calculate lower limb joint angle from kinematics data with Plug-in Gait [17]. Sixteen reflective markers (diameter 14 mm) were attached on the anatomical landmarks of both the left and right lower limbs at the following areas: anterior-superior iliac spine, posterior-superior iliac spine, lateral midthigh, lateral knee, lateral midshank, lateral malleolus, second metatarsal head, and calcaneus. In this study, we analyzed the motions of the right leg stance phase when participants play the backcourt forehand clear stroke actions. The kinematic analysis computed all angular positions of lower limb joint movements, and the plantar pressure analysis computed all peak pressure, contact area, and pressure-time integral.

### 2.4. Data Analysis

All statistical results of each trial were analyzed with the SPSS17.0 (SPSS Inc., Chicago, IL, USA). An independent sample *t*-test was applied for analyzing the differences of the plantar pressure and lower limb kinematics data between the two groups. Cohen's *d* effect size with 90% confidence intervals (CI) was used to present the magnitude of the variables that were significantly different after statistical analysis. Cohen's *d* effect size was interpreted with the following standards: 0–0.19 trivial, 0.2–0.59 small, 0.6–1.19 moderate, 1.2–1.99 large, and ≥2 very large [21]. Statistical significance was set at 0.05. The variations of the three trials for each condition were averaged, and statistical tests were done based on the averages of the six trials. The plantar surface was divided into nine regions, namely, medial rearfoot (MRF), lateral rearfoot (LRF), medial midfoot (MMF), lateral midfoot (LMF), first metatarsal head (FM), second and third metatarsal heads (SATM), fourth and fifth metatarsal heads (FAFM), big toe (BT), and other toes (OT) [9].

## 3. Results

**Figure 2:**
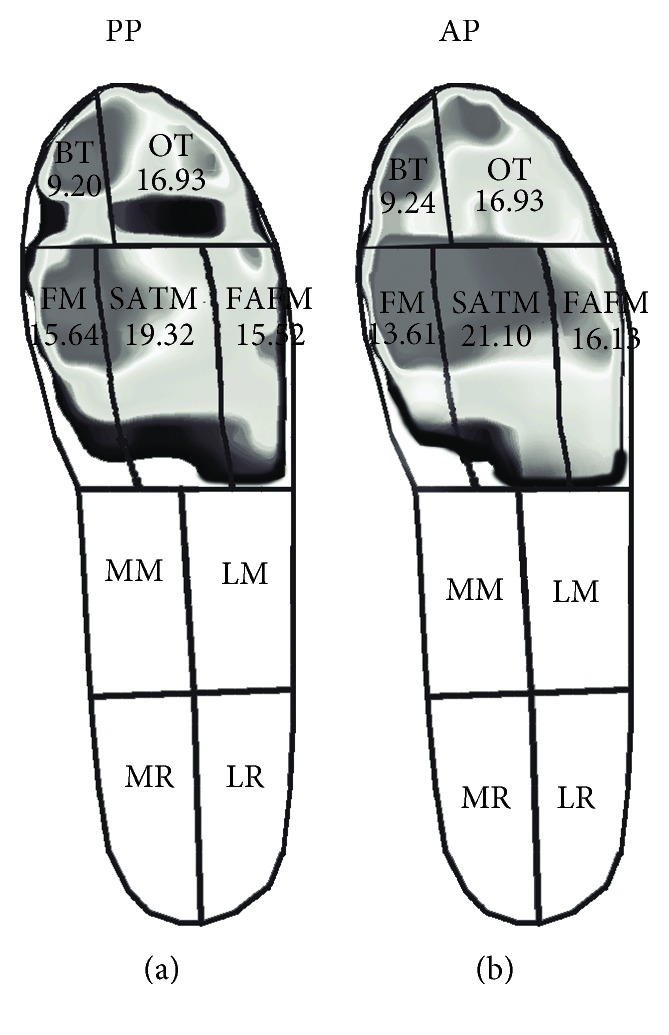
The contact area of the PP (a) and AP (b). Unit: cm^2^.

**Figure 3:**
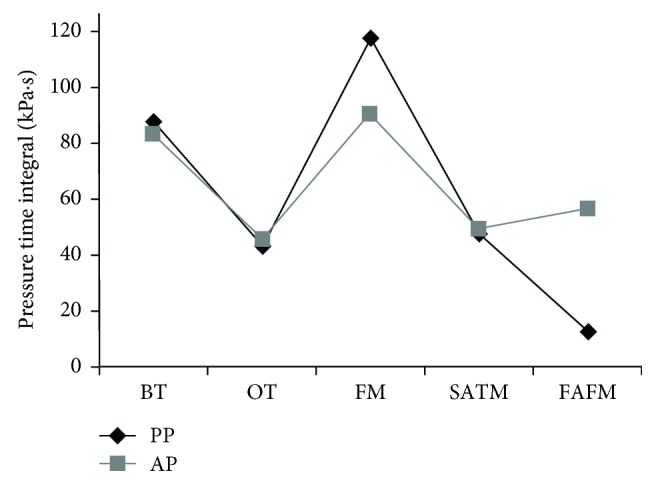
The pressure-time integrals of the AP and PP.

Through the insole plantar pressure measurement, it was found that the contact area, the pressure-time integral, and the force-time integral of the AP were different from the PP summarized in Table 1. For the contact area, both of the PP and the AP were in contact with the ground over the toes and metatarsals, without the midfoot and heel (Figure 2). The PP were associated with higher contact area at the FM regions (*P* ≤ 0.001, *d* = 1.74 (90%CI: −3.10 to −0.96)), whereas the AP were associated with higher contact area at the SATM regions (*P* = 0.006, *d* = 1.40 (90%CI: 0.58 to −2.96)). For the pressure-time integral, the PP had the highest pressure-time integral at the FM regions, which was significantly higher than that of the AP (*P* ≤ 0.001, *d* = 3.20 (90%CI: 18.30 to 33.53)). The AP produced the larger pressure-time integral at the FAFM regions compared to the PP (*P* ≤ 0.001, *d* = 10.75 (90%CI: 46.43 to 36.70); Figure 3). For the force-time integral, in general, the PP had a higher force-time integral at the SATM and FM area compared to other fields while the AP had a higher force-time integral at the BT and metatarsal areas. The BT (*P* ≤ 0.001, *d* = 7.97 (90%CI: −48.61 to −38.37)), FM (*P* ≤ 0.001, *d* = 2.65 (90%CI: −25.18 to −11.95)), and FAFM (*P* ≤ 0.001, *d* = 4.41 (90%CI: −36.05 to −23.02)) of the AP were greater compared to those of the PP, while the PP had the greater force-time integral at the SATM compared to the AP (*P* = 0.009, *d* = 1.32 (90%CI: 1.64 to 9.75)).

**Figure 4:**
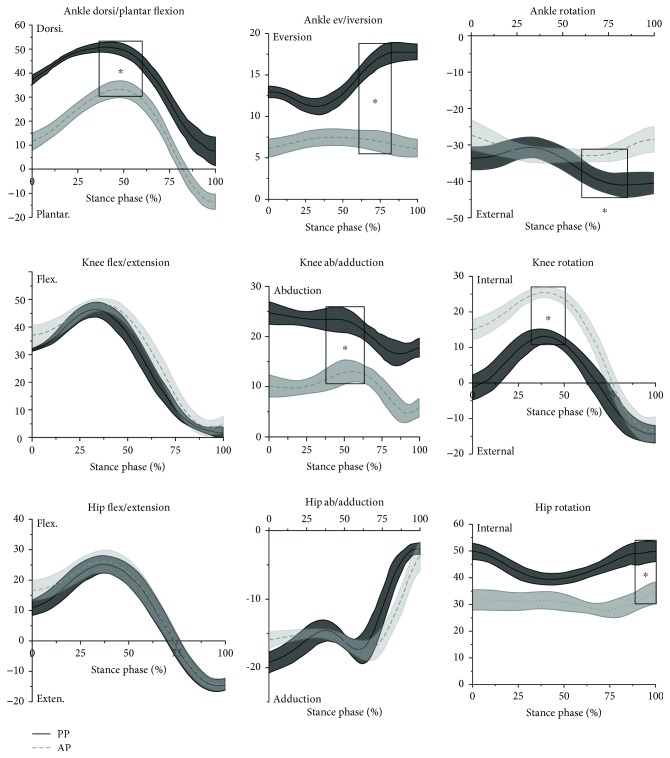
The kinematics of the right lower limb during the stance phase of the forehand overhead clear stroke (statistically significant results were highlighted with rectangles; ^∗^*P* < 0.05).

The kinematics of the right lower limb of participants during the stance phase of the forehand overhead clear stroke is presented in Figure 4. The peak ankle dorsiflexion angle of the AP was significantly lower than that of the PP (*P* ≤ 0.001, *d* = 2.06 (90%CI: 14.88 to 19.68)). The peak ankle eversion angle of the AP was lower than that of the PP (*P* ≤ 0.001, *d* = 6.76 (90%CI: 8.86 to 11.71)). And the peak ankle internal rotation level of the AP was lower than that of the PP (*P* ≤ 0.001, *d* = 3.38 (90%CI: −10.40 to −5.87)). At the knee, the peak knee abduction angle of the AP was lower than that of the PP (*P* ≤ 0.001, *d* = 3.59 (90%CI: 9.42 to 16.39)). And the peak knee internal rotation angle of the AP was bigger than that of the PP (*P* ≤ 0.001, *d* = 8.03 (90%CI: −13.90 to −10.56)). At the hip, the peak hip internal rotation angle of the AP was lower than that of the elite players (*P* ≤ 0.001, *d* = 8.30 (90%CI: 14.90 to 18.00)).

## 4. Discussion

This study focused on exploring the lower limb kinematics and foot pressure in the backcourt forehand clear stroke among badminton players to give theoretical reference in teaching and answer the questions occurring in the process of learning the actions. The results showed that the foot plantar pressure measurement and kinematics among badminton players were significantly different. Accurate determination of touchdown and toe-off events during human locomotion is an important factor for further motion analysis, such as time normalization, to compare specific kinematic and kinetic parameters between participants [22]. In the stroke action of badminton, the stance phase of the lower limb occupies an important position, which directly affects the direction, height, and quality of the jump. Understanding the pressure distribution of the foot could effectively optimize technical action, reduce foot injury, and improve the design of special badminton shoes [16, 23]. The Achilles tendon, plantar fascia, and anterior talofibular ligament were reported as more susceptible to severe injury risks in badminton than in other sports to which the unique and repetitive movements, such as frequent stop-and-go maneuvers required in badminton, contribute to [24]. That highlights the importance to explore the plantar pressure characteristics in badminton movements, but few studies have been undertaken for doing this. Both of the PP and the AP make contact with the ground over the toes and metatarsals, without the midfoot and heel. This is likely due to initial contact with the ground being made during the forefoot strike by all participants during this action who are trying to move as quickly as possible to reduce the time, just like athletes who almost contact the ground with their forefoot to get faster speed during sprinting. This result is consistent with a previous study which verified that the metatarsal heads and lateral foot could be the main contact regions with the surface for different footwork [25]. The PP were associated with higher contact area at the FM regions, whereas the AP were associated with higher contact area at the SATM regions. This might be due to their different pressure distribution patterns as the highest pressure-time integral at the FM region was found for the PP and the larger pressure-time integral at the FAFM regions was identified for the AP.

In this study, the players wore identical pairs of shoes to eliminate the error as the footwear has been also reported to influence plantar pressure measurements [10, 26]. In general, comparable plantar loads were observed between the PP and AP; the plantar pressure analysis indicated that the PP had the highest pressure-time integral at the FM regions, which was significantly higher than that of the AP. The AP produced the larger pressure-time integral at the FAFM regions compared to the PP. Repetitively high plantar pressure is thought to be the potential factor for sports-related injuries in the lower extremity [9, 27, 28]. When designing professional badminton sports shoes, the developers should focus on strengthening the footwear resistance in the central and lateral sides of the metatarsal and forefoot area to meet the requirement of the movement demand, namely, partition the shoe sole with different materials to provide more support and wear resistance. Although it is important to consider the effect of pressure-time integral when considering the potential for injuries such as stress fractures, it is also important to consider the force-time integral. The force-time integral offers valuable insight to evaluate pathomechanics associated with overuse injuries in specific foot regions along the area under stress [29, 30]. For the force-time integral, the PP had the higher force-time integral at the SATM and FM area while the AP had a higher force-time integral at the BT and metatarsal areas. The force-time integral of the AP at the FAFM was greater compared to that of the PP. The abnormal greater force-time integral in the lateral forefoot of the AP might induce the foot ligament injuries and calluses in those areas [14, 24].

The peak ankle dorsiflexion of the AP is lower than that of the PP. The ankle dorsiflexion range is a strong indicator to the risk of ankle sprain [31], and the lower ankle dorsiflexion of AP might be a risk factor for ankle sprain [32]. From Figure 4, we could clearly see that at the end of the stance phase, the AP have a distinctly larger plantar flexion angle compared to the PP. The players with greater ankle plantar flexion could perform a fast side step and efficiently change direction [33]. But other studies pointed out that the great plantar angle of the ankle prior to the flight phase might cause an increased risk of landing damage [34]. The increased touchdown plantarflexion might increase the ankle sprain occurrences [32]. The AP had lower values in ankle eversion and external rotation angles than the PP; the better results of the PP might be due to the stronger muscle strength of their dominant leg as a previous study reported that muscle strength of the dominant leg can be significantly increased by playing badminton year by year [35]. The AP and the PP adopted a similar flexion pattern at the knee. Large knee bend and extensor group of combat load affect the overall coordination of each section of the trunk and upper limbs, while a small knee can block the rotational mobility of the joint. Thus, the reasonable flexion angle of the knee joint is helpful to optimize the technique of the clear stroke. The peak knee abduction angle of the AP is lower than that of the PP. This might be due to the higher knee valgus moment produced by the PP. The PP had the high values in hip internal rotation and contacted the ground with their forefoot; this might be associated with the high knee valgus moment [36]. But the knee internal rotation angle of the AP was larger than that of the PP. At the hip, the peak hip internal rotation angle of the AP is lower than that of the PP. Badminton players always hold rackets using the dominant hand, which leads to asymmetric posture by lateral trunk flexion especially during the racket stroke [17, 37]. So the PP tended to lean their hips more by larger internal rotation.

Some limitations to this study also should be considered. First, only twenty participants participated in this study which means the sample size of this study was limited. Secondly, this study only researched the right leg stance phase of the backcourt forehand clear stroke (right leg) without considering the action of the upper limb and the left leg and the swing phase due to the complexity of this technique. Further study is needed to examine the whole body kinetics chain effect and the swing phase. And to further assess the functional significance of the contribution of the joints of the dominant limb to the stance phase among badminton players, it is necessary to investigate the moments and powers. Thirdly, in this study, the PP were athletes with national second-level certificates, and for higher-level players there might be another case. In addition, in this study the participants were not blind to the condition. Due to the nature of the trial, it was not possible to make use of a blinding protocol.

## 5. Conclusion

Plantar pressure analyses indicated that both of the PP and the AP contacted the ground with the forefoot without the midfoot and heel. In general, the AP produced higher force-time integral than the AP, and the PP had a higher force-time integral at the SATM and FM area while the AP had a higher force-time integral at the BT and FM areas. The work suggests that when designing professional badminton sports shoes, the designers should focus on strengthening footwear resistance in the metatarsal and forefoot area, especially the first metatarsal area, to meet the requirement of the movement demand and take the badminton movement characteristics in different regions of the design. The peak ankle plantar flexion, eversion, and external rotation angle levels of the AP are lower than those of the PP. It is important for the AP group to enhance their ankle strength which can prevent injury and improve performance.

## Figures and Tables

**Table 1:** The contact area, pressure-time integral, and force-time integral for AP and PP.

Plantar regions	PP	AP	*P* value	*d* (95% CI)
Contact area (cm^2^)				
BT	9.20 (±0)	9.24 (±0)	1.0	NaN (0 to 0)
OT	16.93 (±0)	16.93 (±0)	1.0	NaN (0 to 0)
FM	15.64 (±0.471)^a^	13.61 (±1.53)	0.001	1.74 (−3.10 to −0.96)
SATM	19.32 (±0.99)	21.10 (±1.49)^b^	0.006	1.40 (0.58 to 2.96)
FAFM	15.52 (±0.63)	16.13 (±1.01)	0.126	0.73 (−1.41 to 0.19)
Pressure-time integral (kPa^·^s)				
BT	86.17 (±7.29)	83.09 (±6.43)	0.330	0.77 (−19.52 to 1.09)
OT	42.56 (±2.28)	44.32 (±6.30)	0.416	0.37 (−6.22 to 2.69)
FM	115.85 (±8.23)^a^	89.93 (±7.98)	0.000	3.20 (18.3 to 33.53)
SATM	48.32 (±4.88)	49.46 (±4.69)	0.604	0.24 (−5.63 to 3.37)
FAFM	12.44 (±0.98)	56.28 (±3.64)^b^	0.000	10.75 (−46.43 to −36.70)
Force-time integral (N^·^s)				
BT	35.15 (±2.87)	78.62 (±7.16)^b^	0.000	7.97 (−48.61 to 38.37).
OT	37.98 (±5.99)	40.88 (±6.58)	0.358	0.46 (−8.71 to 3.02)
FM	70.65 (±10.94)	89.22 (±6.71)^b^	0.000	2.65 (−25.18 to −11.95)
SATM	55.153 (±3.89)^a^	49.46 (±4.69)	0.009	1.32 (1.64 to 9.75)
FAFM	26.91 (±3.75)	56.45 (±9.22)^b^	0.000	4.41 (−36.05 to −23.02)

Notes: values are expressed as mean (standard deviation). ^a^*P* < 0.05; AP was lower compared to PP. ^b^*P* < 0.05; PP was lower compared to AP.

## Data Availability

The data used to support the findings of this study are available from the corresponding author upon request.
